# An Intelligent Framework for Fault Diagnosis of Centrifugal Pump Leveraging Wavelet Coherence Analysis and Deep Learning

**DOI:** 10.3390/s23218850

**Published:** 2023-10-31

**Authors:** Niamat Ullah, Zahoor Ahmad, Muhammad Farooq Siddique, Kichang Im, Dong-Koo Shon, Tae-Hyun Yoon, Dae-Seung Yoo, Jong-Myon Kim

**Affiliations:** 1 Department of Electrical, Electronics, and Computer Engineering, University of Ulsan, Ulsan 44610, Republic of Korea; niamat016@mail.ulsan.ac.kr (N.U.); zahooruou@mail.ulsan.ac.kr (Z.A.); mfarooq229@mail.ulsan.ac.kr (M.F.S.); 2 ICT Convergence Safety Research Center, University of Ulsan, Ulsan 44610, Republic of Korea; kichang@ulsan.ac.kr; 3 Electronics and Telecommunications Research Institute (ETRI), Daejeon 34129, Republic of Korea; sdk@etri.re.kr (D.-K.S.); thyoon0820@etri.re.kr (T.-H.Y.); ooseyds@etri.re.kr (D.-S.Y.); 4 PD Technology Co., Ltd., Ulsan 44610, Republic of Korea

**Keywords:** centrifugal pump, wavelet coherence analysis, fault diagnosis, convolutional neural network, vibrational signals

## Abstract

This paper proposes an intelligent framework for the fault diagnosis of centrifugal pumps (CPs) based on wavelet coherence analysis (WCA) and deep learning (DL). The fault-related impulses in the CP vibration signal are often attenuated due to the background interference noises, thus affecting the sensitivity of the traditional statistical features towards faults. Furthermore, extracting health-sensitive information from the vibration signal needs human expertise and background knowledge. To extract CP health-sensitive features autonomously from the vibration signals, the proposed approach initially selects a healthy baseline signal. The wavelet coherence analysis is then computed between the healthy baseline signal and the signal obtained from a CP under different operating conditions, yielding coherograms. WCA is a signal processing technique that is used to measure the degree of linear correlation between two signals as a function of frequency. The coherograms carry information about the CP vulnerability towards the faults as the color intensity in the coherograms changes according to the change in CP health conditions. To utilize the changes in the coherograms due to the health conditions of the CP, they are provided to a Convolution Neural Network (CNN) and a Convolution Autoencoder (CAE) for the extraction of discriminant CP health-sensitive information autonomously. The CAE extracts global variations from the coherograms, and the CNN extracts local variations related to CP health. This information is combined into a single latent space vector. To identify the health conditions of the CP, the latent space vector is classified using an Artificial Neural Network (ANN). The proposed method identifies faults in the CP with higher accuracy as compared to already existing methods when it is tested on the vibration signals acquired from real-world industrial CPs.

## 1. Introduction

CPs play a vital role in various industries including engine manufacturing, air conditioning, electricity generation, and chemical processing, with CPs accounting for approximately 70% of all pump types and consuming around 20% of the world’s energy production [1]. Despite their long life span, the sudden failure of CPs can lead to significant disruptions and even catastrophic consequences. These failures result in economic losses, extended costly repairs, and downtime. In order to reduce the potential dangers, it is imperative to regularly monitor the CPs. This monitoring can involve a large workforce or the utilization of signal processing and Artificial Intelligence (AI) techniques, which offer a cost-effective and efficient solution [2]. Recently, AI-based condition monitoring has gained increasing attention, especially for the early detection and diagnosis of CP faults. Therefore, in this paper, an intelligent framework is proposed for the early detection and diagnosis of faults in CPs, which are prone to various operational concerns due to the rapid rotation of impellers, including issues such as damaged bearings, impeller damage, and cavitation [3]. Defects in CPs can be categorized into fluid-flow-related and mechanical faults (MFs) [4]. Mechanical seal-related defects alone account for 34% of CP defects, while impeller problems can cause mechanical or combined mechanical and flow-related defects [5,6]. Mechanical defects can lead to perilous soft failures, which are difficult to detect due to the gradual decline in the efficiency of CP operation [7]. Early identification of soft flaws in CPs is of primary importance. This study focuses on the early identification of soft flaws in CPs, often caused by Mechanical Seal Hole (MSH), Mechanical Seal Scratch (MSS), or Impeller Fault (IF) issues. To achieve this, a condition-based monitoring system (CBM) is one of the most effective methods. CBMs collect data from machines under various operating conditions, extending machine runtime at a relatively low cost [8]. Mechanical faults, such as IFs, MSHs, and MSSs, can significantly impact the vibration signals emitted by CPs. These faults can make vibration signals irregular and unpredictable. Signal processing techniques such as time, frequency, and time–frequency-domain (TFD) analyses are commonly used for this purpose.

AI-based techniques for fault diagnosis consist of signal preprocessing, features preprocessing, and fault identification and classification tasks [9,10,11]. Sakthivel et al. [12] compared dimensionality reduction techniques for CP defect diagnosis and discovered that Principal Component Analysis (PCA) yielded promising results. PCA extracts principal components that contain information about various machinery malfunction symptoms. However, PCA disregards the estimation of intraclass separability. In addition, information loss is a significant disadvantage of PCA. Contrary to PCA, LDA determines the optimal reduced-dimensional representation by considering interclass scatteredness and intraclass separability, given a sufficiently large labeled dataset. Several variants of LDA, such as the trace ratio LDA [13], the local sensitive discriminant analysis [14], and the robust linear optimized LDA [15], have been proposed in recent decades. Li et al. [16] conducted Particle Image Velocimetry (PIV) experiments to examine the correlation between the internal flow field and external characteristics of a low-specific-speed CP. The focus of the investigation was on energy conversion. The findings of the study on the internal flow of CPs have revealed that the rotating impeller’s intricate secondary flow results in additional energy loss in the blade channels and an intensified wake-jet structure, leading to further losses at the blade’s trailing edge (TE) [17]. Therefore, the primary means of enhancing the energy efficiency of pumps is to minimize the occurrence of secondary flow within the impeller. There are different methods of signal processing, including the Fourier transform (FT), that have proven effective for analyzing stationary signals. However, these methods fail to provide accurate information for nonstationary signals due to the loss of temporal data, though they retain spectral component information [18]. Newer signal processing techniques have been introduced to address this limitation, including the wavelet transform (WT), Short-Term Fourier Transform (STFT), and Stockwell Transform (ST) [19]. The STFT uses fixed sample windows for time–frequency analysis but faces trade-offs between time and frequency resolution [20]. In contrast, the WT solves the problem of resolution by utilizing bigger windows for lower frequencies and smaller windows for higher frequencies. This provides useful data in both the frequency and time domains (TD) [21]. However, the WT method is sensitive to noise and does not provide phase information for the analyzed signals. The WT has garnered significant attention in recent years for its efficacy in processing nonstationary signals, leading to successful applications in various domains [22]. For example, it has been effectively utilized in bearing condition monitoring [23,24,25], detection of machine tool failure [26], knock and misfire detection in spark ignition engines [23], fault detection in washing machines [27], and monitoring of alternating-current drives [28]. Researchers have proposed different approaches using wavelet transforms in machine condition monitoring. For instance, an energy-based method by Ruqiang et al. [29] used wavelet coefficients to identify defects in rotary machinery. Utilizing a Hierarchical Neural Network for Bearing Fault Diagnosis through Dimensionality Reduction and Classification, Delgado et al. [30] employed a nonlinear manifold learning technique. Xia et al. [31] presented a CNN-based approach using data fusion and feature representation for rotatory machinery diagnosis. Ahmad et al. [32] introduced a three-phase technique involving the Walsh Transform, raw statistical features, and cosine linear discriminant analysis (CLDA) for fault classification in CP vibration signatures. Sajjad et al. [33] proposed a technique for fault classification in CP that involves computing kurtogram spectra, utilizing a convolution encoder, and implementing a linear classifier for fault visualization and classification. Kuang et al. [34] identified the vibration source in mechanical specimens using wavelet coherence and Fourier coherence.

The abovementioned techniques improved the reliability and performance of CPs by enabling early detection and diagnosis of faults. However, there exist several limitations. (i) Techniques concerning TD correlation analysis suffer from background noise. (ii) FT is best suited for stationary signals. However, the vibration signals obtained from CPs under defective conditions are highly complex and nonstationary. (iii) Techniques concerning STFT suffer from spectral leakage due to windowing effects. To address these issues, this paper proposes an intelligent technique for the fault diagnosis of CP based on wavelet coherence and deep learning. Wavelet coherence analysis is a signal processing technique that is used to measure the degree of linear correlation between two signals as a function of frequency. For Wavelet Coherent Analysis, the selection of a healthy baseline signal is important. For this reason, a proper strategy is adopted for the selection of a healthy baseline signal. The wavelet coherent analysis is calculated between the healthy baseline signal and the signal acquired from the CP under different operating conditions, and coherograms are obtained. The coherograms carry information about the CP’s vulnerability to faults. The coherograms are then provided as input to a CNN and a CAE for the extraction of discriminant CP health-sensitive information. The CAE extracts global variations from the coherograms, and the CNN extracts local variations related to CP health. This information is combined into a single latent space. To identify the health conditions of the CP, the latent space is classified using an ANN.

The novelty in this work can be explained as follows. (i) The vulnerability of CPs toward faults is identified by wavelet coherence between a healthy baseline signal and the vibration signals of the CP obtained under different operating conditions. To the best of the author’s knowledge, the potential of wavelet coherence analysis for CP fault diagnosis has not been explored so far in the literature. (ii) CP health-sensitive features are extracted from the coherograms using a CNN and a CAE. The primary contributions of this study can be succinctly described as follows:

1. CP health-sensitive features are extracted from the coherograms. These coherograms visually represent correlation and coherence patterns in CP vibration signals, enhancing feature extraction, aiding interpretation, and enabling more accurate fault diagnosis, thereby improving industrial pump system reliability.
2. The proposed methodology employs CNN and CAE algorithms to extract features from the coherograms. The CAE extracts global variations from the coherograms, and the CNN extracts local variations related to CP health. These powerful deep learning techniques enable the identification and capture of consistent patterns and structures, enhancing feature extraction and improving fault diagnosis accuracy.
3. The proposed fault diagnosis strategy is validated using data obtained from an actual industrial CP test rig. This approach ensures that the results obtained are comparable to those achieved using other fault diagnosis methods.

The present study is organized as follows: Section 2 presents a detailed discussion of the proposed method, while Section 3 describes the experimental setup and the test rig setup for the CP. Section 4 provides a technical background of the proposed method. Section 5 presents the results and discussion, and finally, Section 6 provides a summary of the findings and proposes recommendations for future research.

## 2. Proposed Approach

**Figure 1:**
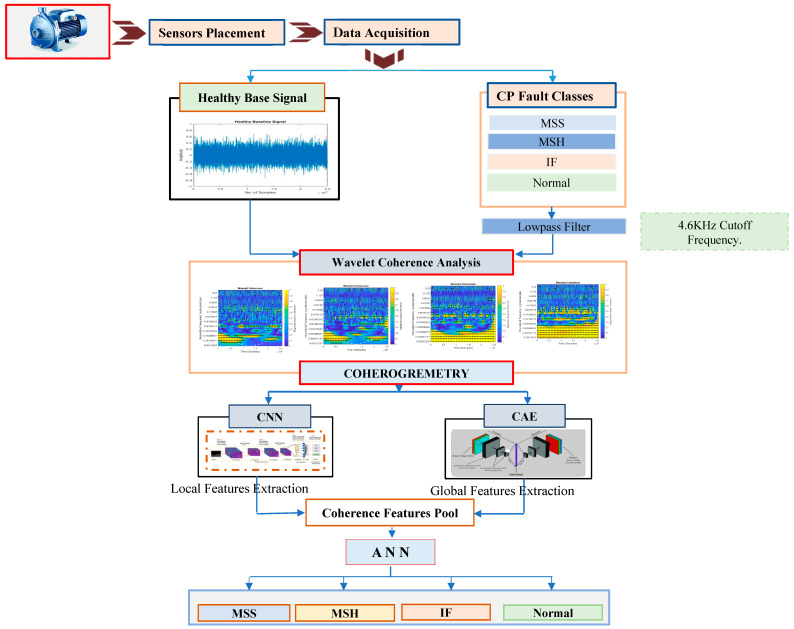
The proposed computationally intelligent framework for CP fault diagnosis.

The proposed method for categorizing faults in a CP involves a combination of signal processing and DL techniques. Initially, to isolate fault-specific frequencies, the sensor is attached to the CP and acquires a vibration signal that is preprocessed through the use of a low-pass filter with a cutoff frequency of 4.6 kHz. Following the preprocessing step, wavelet coherence analysis is applied to convert the 1D signals to 2D signals to find the time-localized oscillation in the signals. We attain the coherent images as an output. The visual representation highlights coherence patterns and structures within the signals, aiding in interpretation and providing a convenient visualization of frequency component relationships according to the health state of the CP, a representation known as a coherogram. Subsequently, a CNN for local features and a CAE for global features are employed to extract CP health-sensitive features from the coherograms, creating a feature pool. These features are then fed into an ANN model to convert the feature image to a feature vector and then utilize these features to identify the ongoing health conditions of the CP (normal, IF, MSH, and MSS). Figure 1 shows an abstract diagram that illustrates the proposed approach.

The steps involved in the proposed approach are as follows:

(1) Using a data acquisition system, the healthy baseline signal and vibration signals of the CP under different operating conditions (normal, IF, MSH, and MSS) are acquired.
(2) To obtain fault-specific signals, we apply a low-pass filter with a cutoff frequency of 4.6 kHz.
(3) Conduct a wavelet coherence analysis on the healthy baseline and the vibration signals of the CP obtained under different operating conditions to assess the correlation and coherence among various frequency components. This analysis identifies the strength and relationships between these components.
(4) Coherogram images are obtained from the wavelet coherence analysis. This visual representation highlights coherence patterns and structures within the signals, aiding in interpretation and providing a convenient visualization of frequency component relationships according to the health state of the CP.
(5) The coherograms carry information about the CP’s vulnerability towards faults as the coherence patterns in the coherograms change according to changes in the health conditions of the CP. In this step, the coherograms are provided to a CNN and a CAE for the extraction of discriminant CP health-sensitive features. The CAE extracts global variations from the coherograms, and the CNN extracts local variations related to CP health. This information is combined into a single latent space. To identify the health conditions of the CP, the latent space is then classified using an ANN.

## 3. Technical Background

### 3.1. Health/Fault-Based Signals

The selection of healthy baseline signals plays an essential role in wavelet coherence analysis. In this study, therefore, an appropriate approach is adopted to select healthy baseline signals. The steps involved in selecting healthy baseline signals are as follows.

#### 3.1.1. Finding the Pump Maximum Efficiency Point

**Figure 2:**
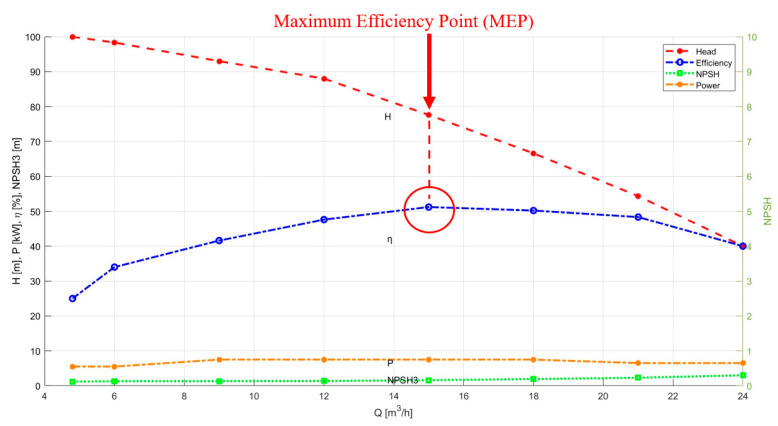
PMT-4008 pump characteristics curve.

The determination of the pump’s maximum efficiency point (MEP) is essential to optimize pump performance. This value helps to determine the flow rate and the net positive suction head (NPSH) of the pump that is most effective. With the flow rate change, the head, energy consumption, and efficiency of the pump also change, producing a characteristic curve. The MEP is the point at which the pump performs most efficiently on this curve. Figure 2 shows the characteristic curve and MEP of the PMT-4008 pump.

The efficiency was determined by evaluating the head, power, and flow through the use of Equation (1):

(1) $$\eta =\frac{Q\rho gH}{P}$$

where *Q* represents the flow rate, *g* is the acceleration due to gravity, and ρ is the density of the fluid being pumped. In addition, the pump’s power consumption can be measured by the variable *P*. Once the pump’s maximum efficiency point has been determined, it can be operated at this point to obtain vibration signals. In this case, twenty healthy vibration signal samples were obtained at the MEP, called the MEP samples.

#### 3.1.2. Healthy Baseline Signal Selection

**Figure 3:**
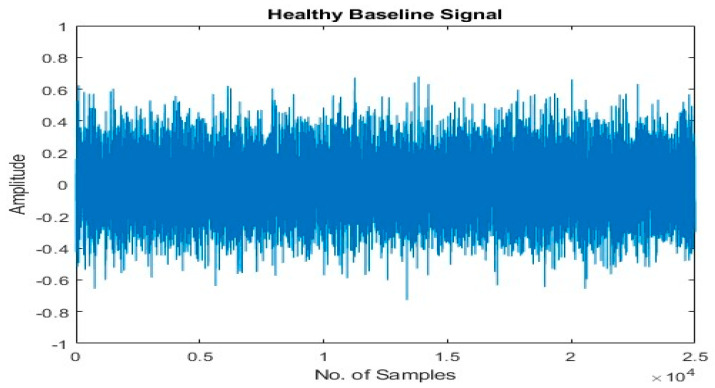
Healthy baseline signal.

After conducting a thorough examination, it became apparent that the most optimal healthy baseline signal from the MEP samples should exhibit a sample mean similar to the mean of the pump’s healthy vibration signals, while also being the furthest from the sample means of other classes, such as MSH, MSS, and IF. Our experiments have demonstrated that this criterion for selecting the healthy baseline signal significantly improves accuracy. However, randomly selecting a healthy baseline signal leads to a decline in classification accuracy. It is crucial to emphasize that the pump’s healthy vibration signals are only obtained under normal operating conditions. The healthy baseline signal selected based on this criterion is depicted in Figure 3.

### 3.2. Wavelet Coherent Analysis

**Figure 4:**
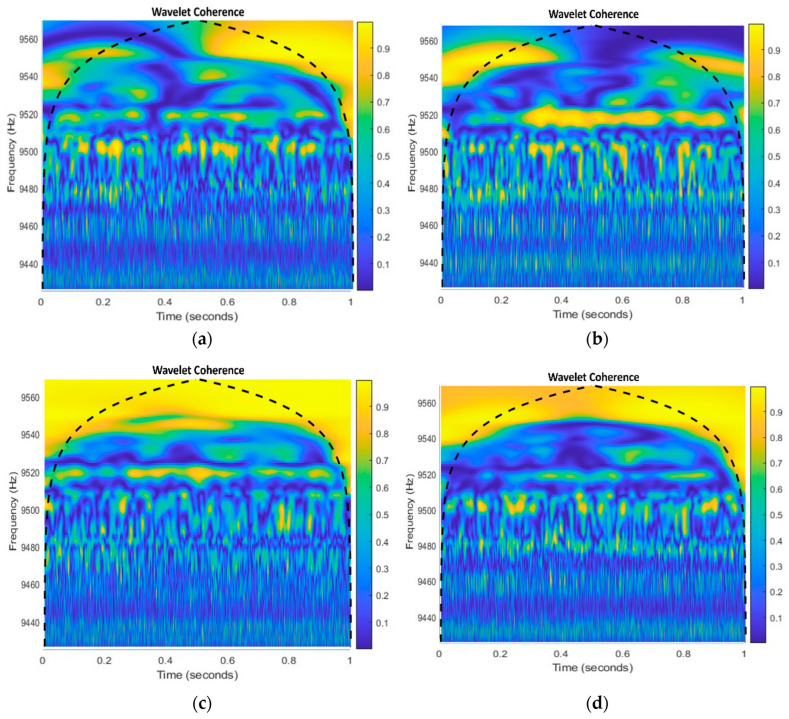
Coherograms of (**a**) an impeller fault, (**b**) a mechanical seal scratch, (**c**) a mechanical seal hole, and (**d**) a normal.

Wavelet coherence analysis is a signal processing technique used to analyze the coherence between two sets of time-domain signals across their entire frequency domain. This method can be beneficial when dealing with vibration signals from a centrifugal pump or similar machinery [35]. The visual representation is shown in Figure 4.

Vibration signals are collected from the centrifugal pump under various conditions. These conditions include the healthy baseline state and different fault classes, representing specific types of pump issues (e.g., IF, MSS, MSH, NC). The collected signals are represented as *xs*1(*t*) as a healthy baseline and *xs*2(*t*) as faulty CP classes, representing the pump’s vibration behavior.

To analyze the coherence between *xs*1(*t*) and *xs*2(*t*), the wavelet transform is applied. The wavelet transform is a mathematical technique that decomposes a signal into its frequency components and their variations over time. In this case, it helps in understanding how the vibration signals change across different frequency components.

(2) $$Wx\left(a,\tau \right)=\frac{1}{\sqrt{a}}\int_{-\infty }^{+\infty }x\left(t\right)\varphi \left(\frac{t-\tau }{a}\right)dt$$

where *a* is the scaling factor, *τ* is the translation factor, and *φ*(*t*) is the Morlet wavelet function.

The cross-wavelet transform is a specific application of the wavelet transform in which the coherence between *xs*1(*t*) and *xs*2(*t*) is examined. It quantifies the similarity and synchronization between these signals in frequency and time domains. The equation for the cross-wavelet transform is:

(3) $$Wxs1-xs2\left(a,\tau \right)=W_{xs1}^{*}\left(a,\tau \right).W_{xs2}^{*}\left(a,\tau \right)$$

$W_{xs1}^{*}\left(a,\tau \right)$ is the complex conjugate of the wavelet transform of $xs1\left(t\right).$

$W_{xs2}^{*}\left(a,\tau \right)$ is the complex conjugate of the wavelet transform of $xs2\left(t\right)$.

The coherence function measures the degree of linear correlation between two signals as a function of frequency. The coherence function varies between 0 and 1, with 0 indicating no correlation and 1 indicating perfect correlation. The coherence between $xs1\left(t\right)$ and $xs2\left(t\right)$ is calculated using the absolute value of the cross-wavelet power spectral density:

(4) $$Coherence=\frac{\left|Wxs1-xs2\left(a,\tau \right)\right|}{\sqrt{Wxs1\left(a,\tau \right).Wxs2\left(a,\tau \right)}}$$

This equation measures the degree of similarity and synchronization between the two signals. Higher values indicate greater coherence. Noise and fault signals are often present in machinery vibration data. Due to the fault’s random and unstable nature, the cross-wavelet power spectral density between fault and normal signals will be minimal, leading to low coherence values.

In order to visually observe the coherence of the whole frequency band and the frequency band of noise and fault signal, the wavelet coherence spectrum of $xs1\left(t\right)$ and $xs2\left(t\right)$ is formed. In the wavelet coherence spectrum, the brightness of the color expresses the coherence. When diagnosing IF in centrifugal pumps using this spectrum, yellow regions indicate operational normalcy, blue regions highlight discrepancies, and green regions suggest transitional dynamics. Yellow reveals typical behavior for MSH issues, blue uncovers seal-hole influences, and green implies minor disturbances. When analyzing MSS, yellow signifies consistency despite the MSS, blue flags disturbances, and green hints at evolving impacts. Even in normal pump operation, yellow shows synchronization, blue may reflect minor fluctuations, and green represents transitional dynamics. However, these color-coded insights guide maintenance and monitoring efforts, focusing on blue regions for timely interventions and repairs in faulty conditions. However, the dashed line in each coherogram represents the cone of influence (COI), which is a region of the time–frequency plane where edge effects can distort the wavelet transform.

The COI is a common feature of wavelet transforms and is used to indicate the regions of the time–frequency plane where the wavelet transform is less reliable. In the coherogram visuals in Figure 4, the COI is represented by the dashed line and indicates the regions of the time–frequency plane where the coherence values may be less accurate due to edge effects.

### 3.3. CNN for Local Feature Extraction

**Figure 5:**
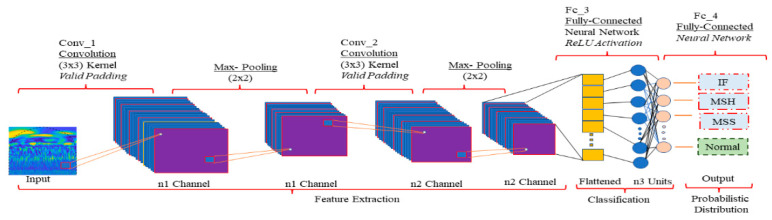
A fundamental structure of a CNN.

CNNs are ideal for local feature extraction. They can learn hierarchical features directly from data, starting with convolutional layers that use learnable filters to identify local patterns such as edges, textures, and colors. As data progress deeper into the network, successive layers extract more complex features [36]. The use of pooling layers between convolutional layers helps to reduce the spatial dimensions of the data, leading to translation and scale invariance. All of this makes CNNs an effective tool for discerning intricate patterns that may be difficult for human observers or traditional algorithms to identify. Figure 5 illustrates the fundamental organizational structure of a CNN.

The CNN model is composed of two main stages: feature extraction and classification. The feature extraction process involves three layers: convolutional, activation, and pooling. These layers use a mix of linear and nonlinear methods to derive representative features from input data. To create a deep CNN, multiple levels of feature extraction are stacked together. The classification stage uses a multilayer perceptron with densely connected layers to classify the input data. Each layer generates a collection of matrices called feature maps that serve as inputs and outputs. The feedforward calculation process is expressed formulaically.

(5) $$f\left(X\right)=f_{L}\left(\ldots \;f_{2}\left(f_{1}\left(X,w^{1}\right),w^{2}\right),\ldots \right),w^{L}\;$$

Here, X represents the input characteristic, such as a piece of text, an image, or a vibrational signal received from the sensor; $w^{1},\;w^{2},\;\ldots \;w^{L}\;$ are the trainable parameters, i.e., weights and biases; $f_{1}\;,f_{2},\;\ldots \;f_{L}$ employ either linear or nonlinear activation functions in corresponding layers. The function *f*(*X*) involves multiple activations, convolutions, and pooling operations on a collection of input features denoted as *X*, with the aim of generating a predicted label.

In Table 1, the CNN initiates with a convolutional layer containing 16 filters of size 3 × 3. These filters traverse the input image, calculating dot products at different locations. This process results in localized feature maps measuring 224 × 224, where each map encapsulates information about specific local characteristics such as edges, corners, or textures. By employing a Rectified Linear Unit (ReLU) activation function, negative values are set to zero, introducing nonlinearity and preserving only relevant features. Following this, a pooling layer with a 2 × 2 filter is applied, leading to a down-sampling effect. This diminishes the image’s spatial dimensions by half, resulting in feature maps of size 112 × 112. This reduction in spatial dimensions serves two primary purposes: it decreases computational complexity and retains the most crucial local attributes, aiding in pattern recognition. As the CNN advances through successive cycles of convolutional, activation, and pooling layers, the spatial dimensions of the feature maps continue to shrink, while the number of filters increases. This hierarchical progression amalgamates local features into more intricate, high-level representations. This aggregation empowers the network to discern complex patterns and structures within the image. Ultimately, the high-level features distilled from these layers are flattened and directed to a fully connected (linear) layer comprising 512 output features. This step marks the transition from feature learning to classification. To counteract overfitting, a dropout layer is introduced, randomly deactivating a proportion of input units during training. The final output layer of the CNN, another linear layer, generates a four-dimensional vector. This vector potentially signifies the probabilities of belonging to four distinct classes in a classification task.

### 3.4. CAE for Global Feature Extraction

A CAE is a neural network architecture primarily employed for unsupervised learning tasks, specializing in the extraction of global features and dimensionality reduction. Comprising an encoder and a decoder, the CAE operates through a sequence of stages. The encoder utilizes convolutional layers to convolve and down-sample the input data, effectively capturing overarching features [37]. These global features are then compactly encoded into a latent representation. The subsequent decoder, utilizing deconvolutional layers, skillfully reconstructs the original data, preserving the essential aspects. Notably, the CAE strives to minimize reconstruction loss, effectively acquiring the ability to represent input data in a manner that accentuates pertinent details while discarding noise and extraneous particulars. The encoder–decoder interplay within the CAE facilitates the discovery of hierarchical features, metamorphosing raw input data into a profound and lower-dimensional representation [38]. This acquired representation, characterized by its focus on global features, can be effectively harnessed for diverse applications, encompassing denoising, feature visualization, and downstream supervised learning endeavors.

In Table 2, in the CAE, the process diverges. Commencing with a convolutional layer equipped with 16 filters of size 3 × 3, these filters operate similarly by scanning the input image. However, their purpose here is to recognize global features instead of localized ones. The calculated dot products assemble global patterns that are indicative of more comprehensive aspects of the input image. With the integration of ReLU activation, negative values are suppressed, favoring relevant information. As the CAE progresses through additional convolutional layers, each featuring a ReLU activation, the network progressively abstracts higher-level global features. This hierarchical refinement aids in capturing the holistic structure of the input image. Following the initial phase of feature extraction, the CAE takes an intriguing turn. It employs transposed convolutional layers (also known as deconvolutional layers) to reverse the process. These layers enlarge the feature maps, restoring the image’s spatial dimensions. This intricate mechanism enables the network to recreate the input image from the extracted global features. In the final layer, a Tanh activation function normalizes the pixel values of the generated image to the range [−1, 1], resulting in a reconstructed image that should ideally resemble the original input. This way, the CAE not only identifies global features through its convolutional layers but also exploits its transposed convolutional layers to reconstruct the input image from the extracted features. The process essentially learns a compact representation of the input image in its latent space, facilitating various applications such as image denoising and compression.

### 3.5. Coherogram Feature-Based ANN Classification with CNN and CAE

**Figure 6:**
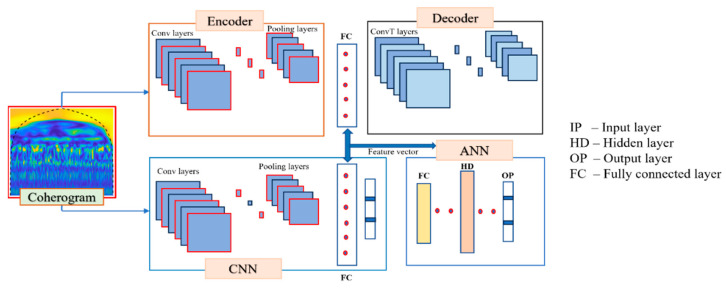
Flow visualization for feature extraction and classification using ANN.

A coherogram is added as input for CNN and CAE to capture local and global features from the coherograms. Local and global feature extraction serve different purposes in image analysis. Local feature extraction focuses on capturing fine-grained details within small image regions, making it suitable for tasks like object recognition and texture analysis. Global feature extraction, as seen in the provided CNN and CAE architectures, aims to capture high-level information and context from the entire image, making it ideal for tasks like image classification and scene recognition. The utilization of parallel CNN and CAE models is justified by their respective strengths: CNNs excel at global feature extraction, while CAEs can compress features and remove noise, making them complementary for tasks that require a combination of high-level context and feature compression. It comprises convolutional and pooling layers designed to extract important features from the input data. Subsequently, a fully connected layer followed the convolution and pooling layers, facilitating the flattening of these features and ultimately leading to an output layer for further processing. In the same manner, global features were extracted from the coherograms of vibrational signals using a CAE. The CAE leveraged the autoencoder’s natural compression capability to derive valuable features that could later be used to reconstruct the original image. The encoder part of the CAE was composed of a sequence of convolutional layers followed by pooling layers, culminating in a fully connected layer. On the other hand, the decoder section of the CAE comprised a fully connected layer combined with a set of transposed convolutional layers responsible for reconstructing the latent space features. In CNNs, FCs are used for high-level feature learning and classification/regression, and in CAEs, FCs are part of the decoder, reconstructing input data from a compressed latent space. For ANN, the features are taken from the FCs of both CNN and CAE and are merged in a feature vector as input for ANN. ANN is designed to categorize input features into their respective classes, consisting of three layers: the input layer, the hidden layer, and the output layer. In the input layer, the ANN takes the input features. The hidden layer carries out a linear transformation of these features through matrix multiplication operations. The detailed illustration of features extractions is shown in Figure 6.

### 3.6. ANN

The process of training an ANN entails the modification of the network’s weights and biases to reduce the loss function through the utilization of backpropagation [39]. During this stage, the ANN is presented with the training data, and the weights are adjusted to minimize the difference between the predicted output and the actual output. The selection of an optimization algorithm, such as stochastic gradient descent (SGD), Adam, or Adagrad, may have an impact on the training procedure. Throughout the training process, it is possible to evaluate the performance of the ANN by utilizing various metrics, including accuracy, precision, recall, and F1 score.

Upon completion of training and evaluation, the ANN can be applied to classify test data. The evaluation of the ANN’s performance on the testing dataset can serve as an estimation of its overall generalization performance. Upon achieving satisfactory performance, the ANN can be utilized for the classification of unseen data.

In Table 3, the ANN architecture designed for CP fault diagnosis comprises three layers. The first layer, “Linear-1,” consists of 256 fully connected units with ReLU activation, offering over 3 million parameters. Following this, “ReLU-2” applies ReLU activation to its 256 inputs but does not introduce any additional parameters. Finally, the “Linear” layer with four output units, housing 1028 parameters, likely serves as the output layer for classifying or regressing CP-related faults based on intricate input features. This architecture appears tailored to handle complex diagnostic tasks within the realm of CP fault diagnosis.

## 4. Experimental Setup and Data Acquisition

**Figure 7:**
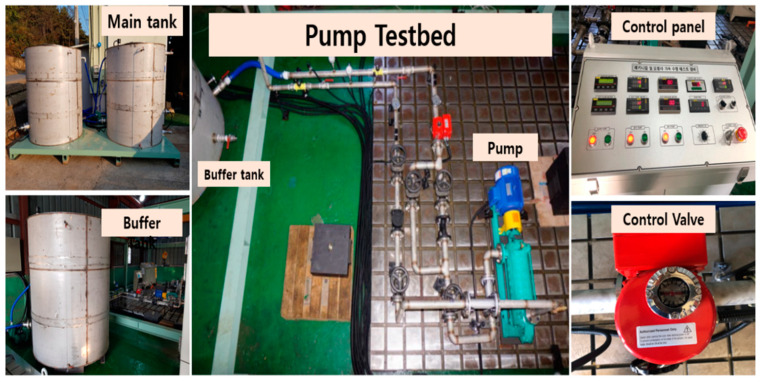
Experimental setup for collecting vibration data.

**Figure 8:**
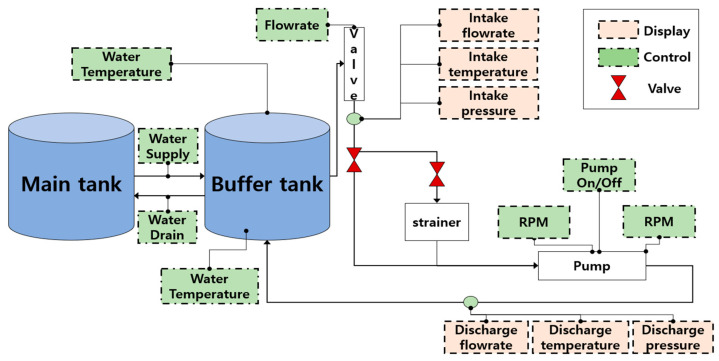
Schematic diagram of the experimental test bed.

The PMT-4008, a frequently utilized pump in the industry, has been designed for experimental purposes. It is equipped with a 5.5 kW motor, a control panel featuring an ON/OFF switch, a speed controller, a flow rate controller, a temperature controller, a water supply controller, pressure gauges, display screens, clear steel pipes, and two tanks (buffer tank and main tank). Figure 7 and Figure 8 depict the experimental setup and a diagrammatic representation of the system, respectively. After the primary setup was established, the test rig was operated to facilitate water circulation within a closed loop from the main tank to the buffer tank. The CP vibration data were recorded using four accelerometers. Two accelerometers were affixed to the pump casing, while the other two were placed near the impeller and the mechanical seal. Each sensor utilizes individual channels to capture the vibration of the pump. 

The data were then transmitted to a signal monitoring apparatus, where they were processed by a National Instruments 9234 device to digitize them. The details of the data collection are listed in Table 4.

Data are collected for a duration of 300 s at a sampling rate of 25.6 kHz. A dataset comprising 1200 samples, each with a length of 25,600, was collected from the CP across different operational scenarios. The present investigation involved the operation of the pump in both typical and simulated faulty conditions. The faults that have been simulated encompass:

- Mechanical seal fault.
  - MSH.
  - MSS.
- Impeller fault.

The signals were obtained through a process of individually simulating each of these faults. The measurement noises pertaining to the gathered signals in each scenario were evaluated in relation to a vibration signal that is considered normal and free from any anomalies. The noise levels corresponding to MSSH, MSS, and IF were determined to be −69.10 dB, −62.07 dB, and −63.78 dB, respectively.

### 4.1. Mechanical Seal Fault

The primary factor leading to seal failure is an excessive amount of pressure. A spring or combination of springs is utilized to maintain contact between the rotating component of the mechanical seal and the stationary component, thereby preventing any leakage from the pump during the installation process. The compression of these springs necessitates a precise amount of pressure. When the pressure exceeds a certain threshold, the mechanical seal faces an excessive amount of pressure. Consequently, this phenomenon has the potential to induce excessive heat, leading to the transformation of the slender film of liquid lubricant situated between the sealing surfaces into a gaseous form. Dirt poses a significant challenge to the mechanical seal. The heightened spring pressure in the absence of a lubricant coating can result in the formation of holes, scratches, and brittleness in the seal faces if any dirt particles become lodged between them during operation. Premature seal failures can have severe consequences, leading to catastrophic pump failure. This study involves the introduction of scratch faults and holes in a mechanical seal, followed by recording vibration signals. This investigation aims to mitigate the negative effects of premature seal failures.

#### 4.1.1. Mechanical Seal Hole

**Figure 9:**
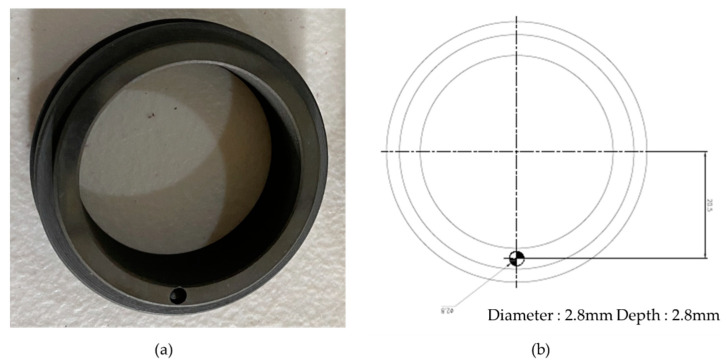
(**a**) Mechanical seal hole. (**b**) Schematic diagram of the mechanical seal hole.

A mechanical seal comprises two constituent parts: a stationary seal and a rotating seal. This study utilizes a pair of seals, both possessing a diameter of 38 mm. The observation in Figure 9 indicates that the rotating component of the two seals exhibited a perforation, while the stationary part of the seals remained free from any imperfections. The aperture exhibited a diameter of 2.8 mm and a depth of 2.8 mm. The aforementioned item was employed as an imperfect barrier to examine a seal’s inherent deficiency linked with an MSH fault.

#### 4.1.2. Mechanical Seal Scratch

**Figure 10:**
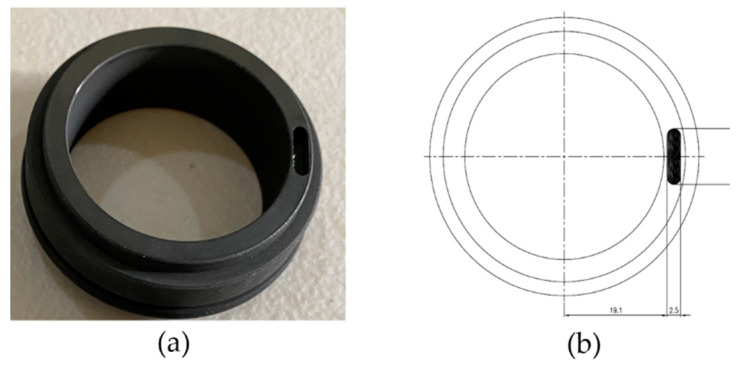
(**a**) Mechanical seal scratch. (**b**) Schematic diagram of the mechanical seal scratch.

The dynamic component of the mechanical seal incurred a surface abrasion, while the static component remained devoid of any defects. The mechanical seal depicted in Figure 10 exhibits a significant defect resulting from a scratch measuring 2.5 mm in diameter, 10 mm in length, and 2.8 mm in depth.

### 4.2. Impeller Fault

Crevice corrosion is a frequently encountered factor leading to impeller malfunction. Crevice corrosion results in a nonuniform surface featuring multiple superimposed apertures of varying dimensions. As a result of the shear stress exerted on the material, the preexisting holes of diverse dimensions have the potential to develop into substantial cracks, thereby inducing fatigue and ultimately leading to a catastrophic collapse. The present investigation involved seeding an impeller with a similar issue, followed by the collection of vibration signals emanating from the defective impeller.

**Figure 11:**
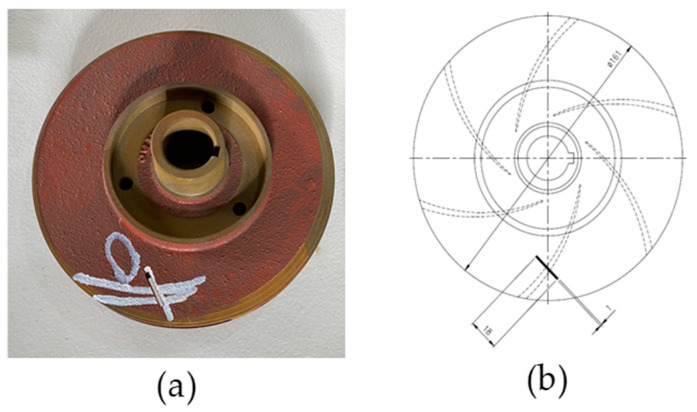
(**a**) Impeller fault. (**b**) Schematic diagram of the impeller fault.

**Figure 12:**
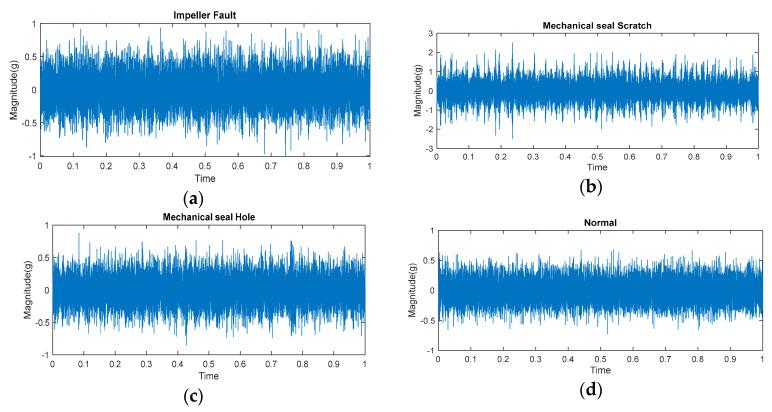
CP vibration signal under different operating conditions: (**a**) an impeller fault condition, (**b**) a mechanical seal scratch condition, (**c**) a mechanical seal hole condition, and (**d**) a normal condition.

This study utilized three cast iron impellers with a diameter of 161 mm. Two impellers were found to be new and in pristine condition. As illustrated in Figure 11, a deficiency was introduced in the third impeller by removing a portion of its metal. The dimensions of the defect were recorded as 2.5 mm in diameter, 18 mm in length, and 2.8 mm in depth. Figure 12 illustrates the vibration signal obtained from the pump under normal and defective operating conditions.

## 5. Results and Discussion

The creation of appropriate training and testing subsets was vital in assessing the effectiveness of the proposed approach for CP health condition identification. In this study, we utilized a dataset of 1200 coherogram images, encompassing 300 images for IF, 300 images for MSH, 300 images for MSS, and 300 images for normal conditions. These images were divided into training and testing sets, with a test size of 0.2. Specifically, the training set was composed of 960 samples, while the testing set consisted of 240 data samples.

### Performance and Comparison

This paper presents an intelligent framework for CP fault diagnosis, combining coherogram analysis with DL. The framework includes key modules for accurate diagnosis and improved feature extraction. The approach uses wavelet coherence analysis to create coherogram images that represent frequency component relationships. DL techniques like CNN and CAE extract discriminant features from these images, which are then fed into an ANN for CP health state identification. This integrated method provides precise fault detection and diagnosis, enhanced feature extraction via coherogram analysis, and efficient fault classification using deep learning and ANN algorithms. Multiple metrics, including accuracy, precision, recall, and F1 score, were utilized to evaluate and contrast the effectiveness of the proposed approach with that of established methodologies. The subsequent enumeration comprises the mathematical Expressions (6)–(9) employed for the computation of said metrics.

(6) $$Accuracy\;\frac{\left(TN+TP\right)}{\left(TN+TP+FN+FP\right)}\times 100\%$$

(7) $$Precision=\frac{TP}{\left(TP+FP\right)}\times 100\%$$

(8) $$Recall=\frac{TP}{\left(TP+FN\right)}\times 100\%$$

(9) $$F_{1}-Score=\frac{2TP}{2TP+FP+FN}=2\times \frac{Precision\times Recall}{Precission+Recall}$$

In terms of classification, TP refers to the true positive instances, which are the samples that have been correctly identified by the classifier. TN, on the other hand, represents the true negative instances, which are the samples that have been incorrectly identified by the classifier. FP denotes the false positive instances, which are the samples that have been incorrectly identified as positive by the classifier, while FN represents the false negative instances, which are the samples that have been incorrectly identified as negative by the classifier.

K-fold cross-validation is a widely used machine learning technique for robustly assessing a model’s performance. Its fundamental principle involves splitting the available data into K subsets or folds. The model is then trained on K-1 of these folds while using the remaining folds for testing. This process iterates K times, ensuring that each fold serves as the test set exactly once. Finally, the results from each iteration are averaged to provide an overall estimate of the model’s performance.

The advantages of K-fold cross-validation are notable. It yields a more reliable estimate of the model’s performance than a single train-test split, mitigating the risk of overfitting by subjecting the model to multiple independent subsets of the data.

In proposed work wisely employed 5-fold cross-validation as a robust evaluation strategy for their DL model, which aimed to diagnose faults in centrifugal pumps. The dataset is divided into 5 subsets, with the model undergoing training on 4 subsets and testing on the remaining one in each iteration. This process was repeated five times, ensuring that all subsets were used for testing exactly once. Subsequently, the results from these five folds were averaged to furnish a comprehensive assessment of the model’s performance.

Upon the application of the suggested technique to the vibrational data collected from a real-world industrial setup, notable improvements were observed in terms of accuracy, precision, recall, and F1 scores, achieving impressive percentages of 99.68%, 98.93%, 100%, and 99.69%, respectively. The outcomes obtained from the proposed method, as compared to the reference methods, are presented in Table 5 for further examination. It is evident from Table 5 that the proposed approach surpassed the reference methods in terms of classification accuracy, exhibiting superior performance. The reasons behind the exceptional performance of the proposed method can be attributed to its fundamental concept of employing WCA and the CNN–CAE for analyzing the vibration signals of the CP. The wavelet coherent analysis is calculated between the healthy baseline signal and the signal acquired from the CP under different operating conditions, and as a result, coherograms are obtained. The coherograms carry information about the CP’s vulnerability to the faults. To utilize this information, the coherograms are then provided to the CNN and the CAE for the extraction of discriminant CP health-sensitive features. The discriminant features helped the classification algorithm ANN for the identification of the health state of the CP with high accuracy. Furthermore, the presented approach offers enhanced time–frequency resolution, increased capability to capture transient events, reduced interference, and simplified interpretation, which collectively contribute to its outstanding performance across all evaluation metrics.

To assess the efficacy of the proposed model, a comparative analysis was conducted with two other relevant models utilized for similar purposes. The first model, employed by Weifang Sun et al. [40] in a comparable experimental setup, utilized a fault diagnosis approach based on converted 2D vibrational signal matrices. They applied the mean curvature algorithm to mitigate interference, employed histograms of oriented gradients (HOG) features for extracting fault characteristics, and utilized a support vector machine for automatic fault classification. Upon implementing the steps outlined by Weifang Sun et al. [40] on our dataset, the method yielded accuracy, precision, recall, and F1 scores of 91.65%, 91.77%, 91.57%, and 91.43%, respectively. The observed underperformance was expected due to the heavy noise influence on the vibrational signals. Moreover, these spectrograms lacked accurate energy distribution information and failed to capture vital phase information within the vibrational signals, contributing to their limited performance. In contrast, the proposed model showcased notable advantages, as it harnessed coherograms, allowing for improved time–frequency resolution, enhanced transient event capture, reduced interference, and ease of interpretation, culminating in significantly higher accuracy, precision, recall, and F1 scores of 99.68%, 98.93%, 100%, and 99.69%, respectively. These outcomes demonstrate the superiority of the proposed model over the referenced approaches, indicating its potential as a promising solution for fault diagnosis in industrial setups.

The second method considered for comparison is the approach introduced by Prosvirin et al. [41], which proposes an end-to-end pipeline for fault diagnosis in CPs using complex time–frequency domain signal analysis techniques. The pipeline involves computing a 1/3-binary tree fast kurtogram to create a two-dimensional representation of vibration signal transients. A CAE and a CNN are then trained to autonomously extract global and local features from the kurtograms, which are merged into a joined feature vector. This vector is fed into a shallow-structured ANN for fault identification. Notably, this method also exhibits limitations in effectively distinguishing weak incipient faults, such as discerning between MSH conditions and normal states. The performance score of the said reference method is, respectively, 95.02%, 87.16%, 91.19%, and 89.22%. After conducting a thorough analysis, it was found that the proposed model had significant advantages over the referenced approaches. One of the key features of the proposed model was the use of coherograms, which allowed for improved time–frequency resolution, enhanced capture of transient events, reduced interference, and made interpretation easier. As a result, the proposed model achieved significantly higher accuracy, precision, recall, and F1 scores, with figures of 99.68%, 98.93%, 100%, and 99.69%, respectively. These results demonstrate the superiority of the proposed model and its potential as a promising solution for fault diagnosis in industrial settings.

**Figure 13:**
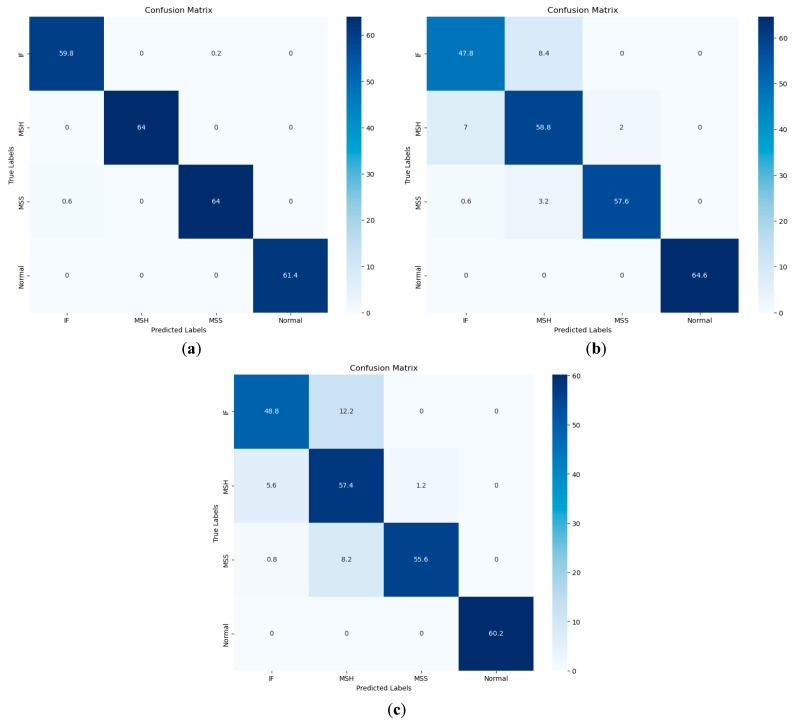
Confusion matrices of (**a**) the proposed model, (**b**) Weifang Sun et al. [40], and (**c**) Prosvirin et al. [41].

The following Table 5 illustrates the comparison of the proposed method with the Weifang et al. [40] and Prosvirin et al. [41] models using our own dataset in terms of all performance parameters for each fault class. Furthermore, Figure 13 describes the confusion matrices comparison of the said models. The confusion matrix plot shows that only the normal class is precisely classified by all models, while the other three classes were only classified precisely by the proposed model.

## 6. Conclusions

This paper presented a methodology for the fault diagnosis of CPs leveraging wavelet coherence analysis and deep learning techniques. The proposed approach consisted of a series of key steps aimed at achieving enhanced feature extraction for accurate fault diagnosis and classification. A healthy baseline signal is selected and then the CP fault signals are acquired. Wavelet coherence is calculated between the healthy baseline signal and the vibration signals of the CP obtained under different operating conditions, which resulted in coherograms. The coherograms provided a visual representation of frequency component relationships, facilitating interpretation and offering a convenient visualization of correlated patterns between the healthy baseline signal and the vibration signals of the CP obtained under different operating conditions. To extract discriminant latent features from the coherogram images, a CNN and a CAE are employed. In the final step, the proposed method provided the latent features to an ANN for the identification of the ongoing health condition of the CP. To assess the performance of the proposed method for diagnosing faults in centrifugal pumps (CPs), a comparison was made with state-of-the-art reference methods. The experimental results demonstrated the effectiveness of the method in detecting and categorizing faults in CPs, achieving an average accuracy of 99.6%, surpassing the accuracy of previous methods, which were 91.65% and 95.02%, respectively. The proposed method is applied in this study to diagnose mechanical defects in CPs. In future research, the method will be further tested to identify fluid-flow-related defects, such as cavitation in CPs.

## Figures and Tables

**Table 1:** The CNN model’s architecture for extracting local features for fault diagnosis in CPs.

Type of Layer	No. of Filters	Kernel Size	Output	Activation Function
Conv2D (1)	16	3 × 3	[−1, 16, 224, 224]	ReLU
MaxPool2D (2)	-	2 × 2	[−1, 16, 112, 112]	-
Conv2D (3)	32	3 × 3	[−1, 32, 112, 112]	ReLU
MaxPool2D (4)	-	2 × 2	[−1, 32, 56, 56]	-
Conv2D (5)	64	3 × 3	[−1, 64, 56, 56]	ReLU
MaxPool2D (6)	-	2 × 2	[−1, 64, 28, 28]	-
Conv2D (7)	128	3 × 3	[−1, 128, 28, 28]	ReLU
MaxPool2D (8)	-	2 × 2	[−1, 128, 14, 14]	-
Linear (9)	-	-	[−1, 512]	ReLU
Dropout (10)	-	-	[−1, 512]	-
Linear (11)	-	-	[−1, 4]	-

**Table 2:** Architecture of the CAE model for global feature extraction.

Type of Layer	No. of Filters	Kernel Size	Output	Activation Function
Conv2D (1)	16	3 × 3	[−1, 16, 112, 112]	ReLU
ReLU (2)	-	-	[−1, 16, 112, 112]	-
Conv2D (3)	32	3 × 3	[−1, 32, 56, 56]	ReLU
ReLU (4)	-	-	[−1, 32, 56, 56]	-
Conv2D (5)	64	3 × 3	[−1, 64, 28, 28]	ReLU
ReLU (6)	-	-	[−1, 64, 28, 28]	-
Conv2D (7)	128	3 × 3	[−1, 128, 14, 14]	ReLU
ReLU (8)	-	-	[−1, 128, 14, 14]	-
Conv2D (9)	255	3 × 3	[−1, 255, 7, 7]	ReLU
ReLU (10)	-	-	[−1, 255, 7, 7]	-
ConvTranspose2D (11)	128	3 × 3	[−1, 128, 14, 14]	ReLU
ReLU (12)	-	-	[−1, 128, 14, 14]	-
ConvTranspose2D (13)	64	3 × 3	[−1, 64, 28, 28]	ReLU
ReLU (14)	-	-	[−1, 64, 28, 28]	-
ConvTranspose2D (15)	32	3 × 3	[−1, 32, 56, 56]	ReLU
ReLU (16)	-	-	[−1, 32, 56, 56]	-
ConvTranspose2D (17)	16	3 × 3	[−1, 16, 112, 112]	ReLU
ReLU (18)	-	-	[−1, 16, 112, 112]	-
ConvTranspose2D (19)	3	3 × 3	[−1, 3, 224, 224]	Tanh
Tanh (20)	-	-	[−1, 3, 224, 224]	-

**Table 3:** Artificial Neural Network (ANN) architecture for CP fault diagnosis.

Layer (Type)	Output Shape	Activation Function	Param #
Linear-1	256	ReLU	3,330,048
ReLU-2	256	-	0
Linear	4	-	1028

**Table 4:** Specifications for data acquisition devices.

Device Name	Specification
Accelerometer (622b01)	Range of Frequency: 0.4 → 10 kHz Sensitivity: 100 mV/g (10.2 mV/g (ms^−2^)) ± 5%
DAQ System (NI9234)	Range of Frequency: 0 → 13.1 MHz Generator: Four analog input channels 24-bit ADC resolution

**Table 5:** Result comparisons of the proposed model with Weifang Sang et al. [40] and Prosvirin et al. [41].

Models	Proposed				Weifang Sun et al. [40]				Prosvirin et al. [41]			
**Fault Type**	**IF**	**MSH**	**MSS**	**N**	**IF**	**MSH**	**MSS**	**N**	**IF**	**MSH**	**MSS**	**N**
**Accuracy (%)**	**99.62**	**100**	**99.10**	**100**	85.68	87.22	93.69	100	93.35	93.00	95.23	98.50
Avg. Accuracy (%)	**99.68%**				91.65				95.02			
**Precision (%)**	**98.93**	**100**	**99.69**	**100**	86.88	83.69	96.50	100	82.30	84.90	83.30	98.15
Avg. Precision (%)	**98.93**				91.77				87.16			
**F1 Score (%)**	**99.27**	**100**	**99.39**	**100**	85.48	87.12	93.69	100	86.58	86.53	86.53	97.25
Avg. F1 Score (%)	**99.69**				91.43				89.22			
**Recall (%)**	**99.62**	**100**	**99.10**	**100**	85.62	84.85	95.25	100	91.30	88.23	88.88	96.36
Avg. Recall (%)	**100**				91.57				91.19			

## Data Availability

Data will be provided upon request.
